# Statin-Induced Thrombocytopenia in a Young Female: A Case Report and Literature Review

**DOI:** 10.7759/cureus.19436

**Published:** 2021-11-10

**Authors:** Muhammad Shehryar, Muhammad Fawad Ashraf, Rana Uzair Ahmad, Sakshi Prasad, Hudson P Franca

**Affiliations:** 1 Division of Research and Academic Affairs, Larkin Community Hospital Palm Springs Campus, Hialeah, USA; 2 Internal Medicine, Mayo Hospital, Lahore, PAK; 3 Internal Medicine, National Pirogov Memorial Medical University, Vinnytsya, UKR; 4 Department of Internal Medicine, Larkin Community Hospital Palm Springs Campus, Hialeah, USA

**Keywords:** statin, drug-induced thrombocytopenia, platelets, refractory thrombocytopenia, atorvastatin

## Abstract

The causal relationship of thrombocytopenia with statin intake has been described in many research articles. Our case discusses the refractory nature of thrombocytopenia in a 22-year-old female, one month following a daily intake of 20 mg atorvastatin. This is the first case ever of drug-induced refractory thrombocytopenia reported in a young patient. We will also discuss previously reported cases of drug-induced thrombocytopenia (DIT) in our manuscript.

## Introduction

One of the family members of the commonly prescribed lipid-lowering drug class - statins, atorvastatin, a 3-hydroxy-3-methylglutaryl-coenzyme A (HMG-CoA) reductase inhibitor, is used in atherosclerotic cardiovascular diseases [1-3].

Its mechanism of action slightly differs from other statins in that it has been shown to play a greater role in decreasing levels of triglycerides to a greater extent [4]. Its most common adverse effects include headache and gastrointestinal distress. However, more serious side effects such as rhabdomyolysis and hepatotoxicity have also been reported [5].

Thrombocytopenia is a blood dyscrasia in which the number of platelets is decreased below a certain level (<150,000 platelets/uL). It may be congenital or acquired. One of the causes of acquired thrombocytopenia is drug-induced thrombocytopenia (DIT). It is thought to be caused by autoantibodies formed against the glycoprotein epitopes of platelets, leading to platelet degradation in the presence of a predisposing drug. This can present with signs such as purpura, bleeding, and easy bruising [6].

Many case reports regarding atorvastatin-induced thrombocytopenia have been published [4,7-10]. All these case reports included middle and old-aged subjects who were taking atorvastatin for cardiovascular purposes. Our case report is unique in that it involves a young female patient who was taking atorvastatin for ‘’weight loss” and developed atorvastatin-induced refractory thrombocytopenia.

## Case presentation

Our case portrays a 22-year-old medical student with a body mass index (BMI) of 34, who presented with complaints of multiple petechiae and ecchymosis all over her body, along with recurrent spontaneous knee joint hematoma for two weeks. She also complained of the formation of bleeding points while brushing her teeth, along with three episodes of epistaxis in the last seven days. Urgent complete blood count (CBC) showed thrombocytopenia with the platelet level at 33,000/uL. RBCs and WBCs were found in the normal range. A peripheral blood film test revealed isolated thrombocytopenia with normal morphology of all the cells and the absence of any atypical presentation. Her genetic history was not significant, with no history of a bleeding disorder. She didn't suffer from any viral infections in the recent past. She wasn't a known case of any chronic illness and wasn't taking anti-platelets or anti-coagulants. She was admitted for further workup for thrombocytopenia.

On further lab tests, her prothrombin time (PT), activated partial thromboplastin time (aPTT), and liver function tests were found to fall in the standard range. The viral panel (Hep B, Hep C, HIV, Epstein-Barr virus (EBV), and Cytomegalovirus (CMV)) and autoimmune factors (antinuclear antibodies (ANA) and rheumatoid factor) were also unremarkable. Hence, a provisional diagnosis of immune thrombocytopenic purpura was made, and she was started on IV-dexamethasone 4 mg TDS and was observed with daily CBC. Her platelet levels improved sharply with levels of 59,000/uL on admission day 3 and 94,000/uL on day five. She started to improve clinically with no petechiae formation on the tourniquet test. Therefore, she was discharged on oral steroids (30 mg oral dexamethasone in three divided doses) with follow-up after three weeks.

Fifteen days after discharge, she presented in the ER with hypovolemic shock after having severe menstrual bleeding (>9 soaked pads in a day) for the last five days. Blood pressure on first contact was 80/40 mmHg with pulse increased to 144. She was resuscitated with IV fluids and an urgent CBC was ordered, which showed Hb at 7 mg/dL and platelets at 4000/uL (re-confirmed manually). She was transfused three pints of whole blood along with IV-dexamethasone, 20 mg, and two doses of IVIG (400 mg/kg) over one day. Her Hb level improved to 9 g/dL after transfusion and menstrual bleeding stopped, but her platelets levels failed to respond and remained at only 5000/uL. Repeat peripheral blood film and PT and aPTT levels were found normal again.

On detailed history, the patient reluctantly admitted to using atorvastatin 10 mg twice a day for the last two months, which, she, being a medical student, self-prescribed for "weight loss" (she didn't give a statin intake history on her first visit). History further confirmed that she restarted her statins following the previous discharge. However, she remained strictly compliant with steroids use during the last 15 days. Considering the sudden and sharp drop of platelets despite strict steroid use in the last 15 days, and temporal relation of atorvastatin-induced thrombocytopenia, a provisional diagnosis of atorvastatin-induced refractory thrombocytopenia was made. Her statin intake was discontinued. She was initiated on IV dexamethasone 4 mg TDS, intravenous immune globulin (IVIG) (400 mg/kg) QD. Her platelet count improved to 40,000/uL on day three and 89,000 on day six. Her clinical condition improved, however, as soon as she tapered off the dose of steroids, her platelet count dropped sharply to <50 k/uL. Considering this refractory nature, she was initiated on eltrombopag 50 mg QD and 375 mg/m^2^, rituximab 4 weekly, and discharged with monthly follow-up. Her platelet values and clinical condition remained stable in the following months. Therefore, IVIG, eltrombopag, and rituximab were discontinued after a month, and steroids were continued for the following three months.

**Figure 1:**
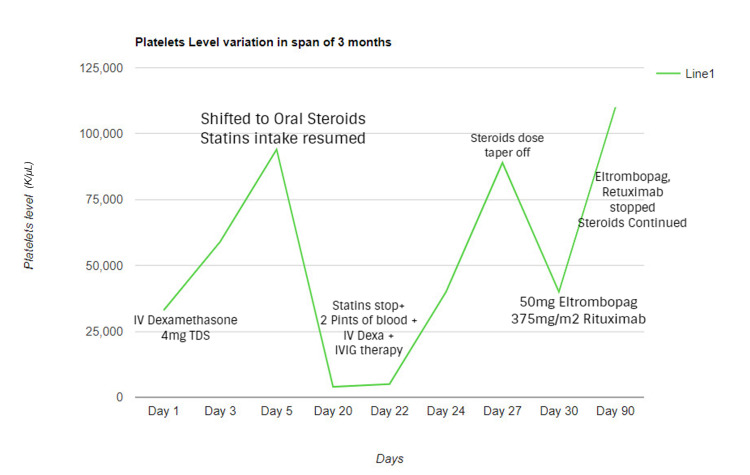
Variations in platelet count in relation to therapy

Platelet level variation over the span of three months is illustrated in Figure 1.

## Discussion

Our patient developed DIT 10 weeks after starting treatment with atorvastatin. Exclusion of other causes of ITP (e.g., post-viral, primary autoimmune conditions) favors the diagnosis of DIT due to atorvastatin. Furthermore, the return of platelet count to baseline levels after discontinuation of atorvastatin supports the diagnosis of DIT. The rest of the general physical and systemic examinations were unremarkable. The drop in the patient’s platelet count after discontinuation of treatment with oral steroids and IVIG suggests that she has a refractory case of DIT [8].

DIT has been shown to be caused by dozens of medications [6]. Two major causes of drug-induced thrombocytopenia have been described in the literature: Bone marrow suppression due to drug administration and increased clearance of platelets from the periphery [11-12].

The cause-effect relationship, in this case, was carried out using the Naranjo Adverse Drug Reaction Probability Scale. The Naranjo algorithm is a standardized questionnaire consisting of 10 questions used to assess the possibility of a drug causing some adverse reactions. Scoring used for the Naranjo algorithm was as follows: >8 = definite adverse reaction; 5-8 = probable adverse reaction; 1-4 = possible adverse reaction, and <1 = doubtful adverse reaction [13-14]. Our patient’s score came out to be 9, which shows a definite association between starting of atorvastatin and the onset of thrombocytopenia as shown in Table 1.

**Table 1:** Naranjo algorithm score for the present case

Question	Yes	No	Do not Know	Score
Are there previous conclusive reports on this reaction?	+1	0	0	Yes, +1
Did the adverse event appear after the suspected drug was administered?	+2	-1	0	Yes, +2
Did the adverse event improve when the drug was discontinued or a specific antagonist was administered?	+1	0	0	Yes, +1
Did the adverse event reappear when the drug was readministered?	+2	-1	0	Yes, +2
Are there alternative causes that could on their own have caused the reaction?	-1	+2	0	No, +2
Did the reaction reappear when a placebo was given?	-1	+1	0	Not Done, 0
Was the drug detected in blood or other fluids in concentrations known to be toxic?	+1	0	0	Not Done, 0
Was the reaction more severe when the dose was increased or less severe when the dose was decreased?	+1	0	0	Not Done, 0
Did the patient have a similar reaction to the same or similar drugs in any previous exposure?	+1	0	0	No, 0
Was the adverse event confirmed by any objective evidence?	+1	0	0	Yes, +1
	Total Score: +9			

To confirm the causal relationship between drug exposure and thrombocytopenia, specific laboratory testing is required, which includes the incubation of control platelets with patient and control sera in the presence and absence of the drug. Such testing is seldom undertaken in routine practice, and there was no facility to perform this test in our laboratory. However, all other results were in favor of drug-induced effects; the major of them being sharp rises after discontinuation of the inducing agent.

Recently reported cases of statin-induced thrombocytopenia have been presented in Table 2. It is to be noted that statin-induced thrombocytopenia is not only associated with atorvastatin but simvastatin and rosuvastatin as well. However, atorvastatin appears to be the most common one to cause this complication. Of all the cases of DIT due to atorvastatin use, only one reported refractory thrombocytopenia [8]. It is also interesting to note that in all these previously reported cases, patients were elderly (mean age = 64yrs), but our study includes a young female of 22 years. Moreover, our patient also developed refractory thrombocytopenia, which is very rare.

**Table 2:** Literature review of statin-induced thrombocytopenia

Reported Case	Age of Patient	Gender	Drug	Dose
Present case	22	Female	Atorvastatin	20mg
Ghuman et al. (2021) [8]	69	Female	Atorvastatin	20mg
Moitra et al (2016) [9]	65	Male	Atorvastatin	10mg
Cvetković et al (2013) [15]	78	Female	Simvastatin	20mg
Narayanan et al (2010) [16]	44	Male	Atorvastatin	20mg
Vrettos et al (2009) [11]	65	Female	Rosuvastatin	Unspecified
Ames et al (2008) [17]	63	Male	Simvastatin	10mg

## Conclusions

Statins have been implicated in multiple cases of drug-induced thrombocytopenia. Our study describes a 22-year- old female who developed thrombocytopenia when she was taking 20 mg/day of atorvastatin for 10 weeks. After the diagnosis, she was treated with oral steroids and IVIG but developed refractory thrombocytopenia requiring treatment with eltrombopag and rituximab. Clinicians should keep DIT high on their differential when a patient presents with thrombocytopenia after starting therapy with statins even if the patient is a young adult. Future researchers should strive to explore the association and effective management of refractory thrombocytopenia secondary to statin therapy.
